# Cognitive performance is related to cortical grey matter volumes in early stages of schizophrenia: A population-based study of first-episode psychosis

**DOI:** 10.1016/j.schres.2009.06.020

**Published:** 2009-09

**Authors:** Taís M. Minatogawa-Chang, Maristela S. Schaufelberger, Adriana M. Ayres, Fábio L.S. Duran, Elisa K. Gutt, Robin M. Murray, Teresa M. Rushe, Philip K. McGuire, Paulo R. Menezes, Marcia Scazufca, Geraldo F. Busatto

**Affiliations:** a Department of Psychiatry, University of São Paulo Medical School, Rua Ovídio Pires Campos, s/n, CEP 05403-010, São Paulo, Brazil; b Department of Psychological Medicine and Psychiatry, Institute of Psychiatry, King's College, De Crespigny Park, SE5 8AF, London, UK; c School of Psychological Sciences, University of Manchester, Oxford Road, Manchester M13 9PL, UK; d Department of Preventive Medicine, University of São Paulo Medical School, Avenida Doutor Arnaldo 455, CEP 01246-903, São Paulo, Brazil

**Keywords:** Cognition, Magnetic resonance imaging, Voxel-based morphometry, Psychosis, Schizophrenia, First episode

## Abstract

**Background:**

Neuropsychological deficits have been reported in association with first-episode psychosis (FEP). Reductions in grey matter (GM) volumes have been documented in FEP subjects compared to healthy controls. However, the possible inter-relationship between the findings of those two lines of research has been scarcely investigated.

**Objective:**

To investigate the relationship between neuropsychological deficits and GM volume abnormalities in a population-based sample of FEP patients compared to healthy controls from the same geographical area.

**Methods:**

FEP patients (*n* = 88) and control subjects (*n* = 86) were evaluated by neuropsychological assessment (Controlled Oral Word Association Test, forward and backward digit span tests) and magnetic resonance imaging using voxel-based morphometry.

**Results:**

Single-group analyses showed that prefrontal and temporo-parietal GM volumes correlated significantly (*p* < 0.05, corrected) with cognitive performance in FEP patients. A similar pattern of direct correlations between neocortical GM volumes and cognitive impairment was seen in the schizophrenia subgroup (*n* = 48). In the control group, cognitive performance was directly correlated with GM volume in the right dorsal anterior cingulate cortex and inversely correlated with parahippocampal gyral volumes bilaterally. Interaction analyses with “group status” as a predictor variable showed significantly greater positive correlation within the left inferior prefrontal cortex (BA46) in the FEP group relative to controls, and significantly greater negative correlation within the left parahippocampal gyrus in the control group relative to FEP patients.

**Conclusion:**

Our results indicate that cognitive deficits are directly related to brain volume abnormalities in frontal and temporo-parietal cortices in FEP subjects, most specifically in inferior portions of the dorsolateral prefrontal cortex.

## 1 Introduction

Neuropsychological deficits have been reported in association with first-episode psychosis (FEP) (Ayres et al., 2007; Bilder et al., 2000; Eastvold et al., 2007). While some studies have highlighted performance deficits in memory (Barch et al., 2001; Barnett et al., 2005), executive functioning (Chan et al., 2006), attention (Nuechterlein et al., 2006) or language (Thomas et al., 1996), a growing body of literature suggests the existence of a diffuse pattern of cognitive impairment in FEP subjects (Lencz et al., 2006; Mohamed et al., 1999).

Many studies have reported brain structural alterations in FEP subjects, most often using magnetic resonance imaging (MRI) with region-of-interest (ROI) based volume measurements (Keshavan et al., 2005; Kubicki et al., 2002; Vita et al., 2006). Ventricular enlargement and whole brain or frontal/temporal regions volume reductions have been the most consistent findings (Flashman and Green, 2004; Steen et al., 2006; Vita et al., 2006).

Investigations using voxel-based morphometry (VBM) have shown reduced grey matter (GM) volumes in neocortical prefrontal and temporal regions, anterior cingulate cortex, insula, hippocampus and parahippocampal gyrus (Chua et al., 2007; Glahn et al., 2008; Honea et al., 2005; Job et al., 2002; Kubicki et al., 2002; Morgan et al., 2007; Schaufelberger et al., 2007). VBM-based techniques avoid the risk of observer-dependent biases associated with manual tracing-based ROI measurements and allow automated whole-brain assessments (Ashburner and Friston, 2000), an advantage for present purposes considering that cognitive deficits may be associated with abnormalities in several brain regions (Wolf et al., 2008).

Few studies have focused on the possible inter-relationship between neuropsychological and brain volumetric research in psychosis. Cognitive performance has been associated with patterns of brain asymmetry (DeLisi et al., 1997; Hoff et al., 1992), whole-brain volumes (Gur et al., 1999b; Zipursky et al., 1998) or ROI-based volumes of the temporolimbic (Gur et al., 2000b; Szeszko et al., 2002) and prefrontal cortices (Gur et al., 1998, 2000a; Szeszko et al., 2000). However, there are studies in which no brain volume–cognition relationship has been found in FEP subjects (Premkumar et al., 2008).

Two studies have investigated the GM volume–cognition inter-relationship in FEP subjects using the VBM approach. Salgado-Pineda et al. (2003) reported a positive correlation between attentional deficits and volumetric abnormalities in thalamic, inferior parietal and frontal regions in 13 FEP subjects. Antonova et al. (2005) showed a significant correlation between generalized cognitive deficits in a sample mixing 41 patients with chronic schizophrenia and 4 FEP subjects and GM reductions within left fronto-temporal regions.

In the context of an epidemiological study investigating the incidence of FEP in São Paulo, Brazil (Menezes et al., 2007), we have reported significant cognitive deficits in a large group of FEP patients relative to asymptomatic controls (Ayres et al., 2007). Using VBM in the same sample, we found significantly decreased GM volumes in the FEP group relative to controls within the left superior temporal and inferior prefrontal cortices, insula bilaterally and right hippocampal region (Schaufelberger et al., 2007).

We report herein the results of an investigation of the GM volume–cognition relationship in the above FEP sample. Given the paucity of previous findings of significant correlations between regional brain volume and cognitive measures in non-elderly healthy subjects (Gur et al., 1999a; Tisserand et al., 2000; Van Petten, 2004), we predicted that significant correlations would emerge only in the FEP sample, indicating specific relationships between localized brain tissue abnormalities and cognitive impairment. In particular, we hypothesized the presence of direct correlations in cortical regions previously shown to present volume abnormalities in association with FEP in VBM studies (Chua et al., 2007; Morgan et al., 2007; Schaufelberger et al., 2007), including the dorsolateral prefrontal region, insula, hippocampus/parahippocampal gyrus, anterior cingulate and temporo-parietal neocortices.

## 2 Materials and methods

### 2.1 Subjects and clinical assessment

A total of 122 FEP subjects and 94 healthy neighbors from the same catchment area underwent MRI scanning (Schaufelberger et al., 2007). All subjects were drawn from a large sample of an epidemiological study of incident cases of psychosis in São Paulo (Menezes et al., 2007; Schaufelberger et al., 2007).

Inclusion criteria for FEP subjects were: age between 18 and 50 years and diagnosis of psychosis according to the Diagnostic and Statistical Manual for Mental Disorders — 4th edition (DSM-IV) (American Psychiatric Association, 1994) criteria (295–298, psychotic codes) as assessed by the Structured Clinical Interview for DSM-IV-SCID (First et al., 1995). Patients with psychotic disorders due to a general medical condition or substance-induced psychoses were excluded. Other exclusion criteria for FEP patients and controls were: history of head injury; neurological disorders or organic disorders affecting the central nervous system; and contraindications for MRI scanning. Control subjects with current or past history of DSM-IV axis I mental disorders, except substance misuse or mild anxiety disorders, were also excluded.

Handedness was assessed with Annett's Hand Preference Questionnaire (Annett, 1970). Diagnostic criteria for substance abuse or dependence were confirmed using the SCID (First et al., 1995). Demographic and clinical data are presented in Table 1.

The neuropsychological assessment included the forward and backward digit span tests from the Wechsler Memory Scale — Third Edition (Wechsler, 1995) and the Controlled Oral Word Association Test (COWAT; Benton and Hamsher, 1978), in order to respectively explore the cognitive domains of: attentional span, working memory and executive functioning (verbal fluency and effortful self-initiation of action). There were two main reasons for our choice of those three specific tasks: their widely known validity to estimate dysfunction on key cognitive domains in schizophrenia (Hutton et al., 1998; Riley et al., 2000; Silver et al., 2003); and their easiness of use in a population-based setting, as it would not have been feasible to apply a broader cognitive in the epidemiological sample from which the data for the groups of psychosis subjects reported herein and their controls were extracted from (Ayres et al., 2007). The average time from the initial case identification due to first-onset psychotic symptoms and cognitive testing equaled 268 ± 393 days in the overall psychosis sample, 350 ± 496 days in the schizophrenia subgroup and 167 ± 164 days in the affective psychosis subjects. In all such groups, the distribution of the length of psychosis until cognitive testing across subjects was continuous rather than bimodal. Fourteen out of the 122 FEP subjects were not assessed with neuropsychological tests because contact was not feasible. A further six FEP patients were excluded due to their inability to read and one refused to take part. All 94 control subjects underwent neuropsychological testing. Due to the variability in the length of time between neuropsychological assessment and MRI scanning, we excluded 13 FEP patients and 8 controls that stood as outliers (i.e. subjects with an interval between the two assessments greater than the 95% confidence interval of the difference between the two assessments). This resulted in a final sample of 88 FEP patients, with a mean length of time between the two assessments equal to 17.27 ± 17.97 days, and 86 control subjects (60.07 ± 55.74 days between the two assessments) (Mann–Whitney-*U*, *Z* = − 6.373, *p* < 0.0001).

From the 88 FEP patients, there were 48 (54.5%) with schizophrenia, 34 (38.6%) with affective psychosis (17 with bipolar disorder and 17 with psychotic major depression) and 6 (6.8%) classified under other psychosis categories (schizoaffective disorder, brief psychosis and psychotic disorder not otherwise specified) according to DSM-IV (APA, 1994). At the time of neuropsychological testing, 33 FEP subjects (37.5%) were not on treatment, whereas 55 (62.5%) were taking antipsychotics (43.2% typicals, 18.2% atypicals and 1.1% both). At the time of MRI scanning, 60 (68.2%) were taking antipsychotics (65% typicals, 33.3% atypicals and 1.7% both), 17 (19.3%) received antidepressant agents, and 15 (17%) were taking mood stabilizers.

Local ethics committee approved the study and all subjects gave written informed consent.

### 2.2 MRI data acquisition

Imaging data were acquired using two 1.5 T GE Signa scanners (General Electric, Milwaukee WI, USA) at the University of São Paulo Clinics Hospital. A series of 124 contiguous axial coronal images was acquired using a T1-SPGR sequence, TE/TR = 5.2 ms/21.7 ms, flip angle = 20°, field of view = 22 cm, matrix = 256 × 192, and voxel dimensions 0.86 × 0.86 × 1.5 mm. A total of 49 FEP subjects and 53 controls were examined using scanner 1, and 39 FEP patients and 33 controls on scanner 2. Intra-class correlation (ICC) coefficients for GM volumes, based on data from 6 healthy volunteers scanned with both equipments, were higher than 0.90 for cortical regions and medial temporal structures (Schaufelberger et al., 2007). Subcortical structures (thalamus and basal ganglia), which showed ICC values lower than 0.85, were excluded from the analysis.

### 2.3 Image processing

Data were processed using Statistical Parametric Mapping software (SPM2) (Wellcome Department of Imaging Neuroscience, London), running in MATLAB 6.1 (Mathworks, Sherborn, MA). Processing details have been described elsewhere (Schaufelberger et al., 2007). Data were processed using optimized VBM routines (Good et al., 2001); images were segmented, spatially normalized, re-sliced using tri-linear interpolation to a final voxel size of 2 × 2 × 2 mm^3^, modulated and smoothed with a 12 mm Gaussian filter.

### 2.4 Statistical analysis

#### 2.4.1 Neuropsychological tests

In order to verify if the scores on the COWAT, forward and backward digit span tests could be combined and analyzed as a single variable representing the neuropsychological performance of the groups studied, a principal component analysis (PCA) was conducted initially, for each separate group (healthy subjects, schizophrenia patients and affective psychosis patients). The factors were selected with varimax rotation and eingenvalues higher than 1. The percentage's cumulative variances were 68.2%, 70.3% and 64.6%, respectively for control subjects, schizophrenia and affective subgroups, with such variance explained by one single factor including the 3 test score variables (COWAT, forward and backward digit scores). We then calculated the Cronbach's alpha value for two summary indices: a composite measure given by the simple sum of the COWAT, forward and backward digit tests results; and the factor score for the first principal component of the linear solution of the PCA. As the best reliability (Cronbach's alpha = 0.77) was obtained for the former (sum of the 3 cognitive test results), we decided to use this measure as a summary index of cognitive performance in the present study.

#### 2.4.2 Correlation between GM volume and cognition

Firstly, separate voxel-based analyses for the groups of psychosis subjects and controls were conducted using the random effects model “single subject: covariates only” in SPM2, including age, gender, years of education and diagnosis of substance misuse as covariates of no interest. The inclusion of such covariates was based on the fact that demographic variables significantly affect both cognitive performance and GM volumes in the general population (Hänggi et al., 2008; Silveri et al., 2008; Zimmerman et al., 2006), as well as our previous population-based findings of a significant influence of comorbid substance abuse on the magnitude of cognitive deficits detected in FEP subjects relative to their neighboring non-psychotic controls (Ayres et al., 2007). The sum of the COWAT, forward digit and backward digit span scores (as a summary cognitive performance index) was entered as the covariate of interest, as well as the total amount of GM, given by the sum of voxels within the GM compartment of each subject. Resulting statistics were thresholded at the one-tailed *p* < 0.001 level of significance (*Z* > 3.09) and displayed as statistical parametric maps (SPMs) into standard anatomical space, with a minimum cluster size of 10 voxels. We used the small volume correction (SVC) approach to conduct a hypothesis-driven investigation of the brain regions where GM abnormalities have been identified in VBM studies of FEP, including the anterior cingulate, insula, hippocampal region, temporo-parietal neocortex and dorsolateral prefrontal cortex (Honea et al., 2005; Schaufelberger et al., 2007). Each region was circumscribed by applying spatially normalized volumes onto the SPMs, based on the anatomical volumes of interest available within the Automatic Anatomical Labeling (AAL) SPM toolbox. Any clusters of voxels showing significant findings were reported if surviving family-wise error (FWE) correction for multiple comparisons at the *p* < 0.05 level, with findings significant at the *p* < 0.10 level (corrected) being reported as trends. The inferior and superior portions of the dorsolateral prefrontal cortex were evaluated using separate volumes of interest, for the following reasons: their distinct contribution to the cognitive operations involved in the tasks used in the present study and to the neuropsychological deficits associated with psychosis (Convit et al., 2001; Suzuki et al., 2005); and our previously reported finding of GM volume deficits circumscribed to the inferior dorsolateral prefrontal cortex (BA 9/45/46) in FEP subjects relative to controls (Schaufelberger et al., 2007). We also conducted an exploratory investigation of significant correlations in other brain regions, using a *p* < 0.05 level corrected for multiple comparisons for the entire brain volume. Such analyses were conducted for the overall FEP group and for each of the diagnostic subgroups (schizophrenia, overall affective psychosis, bipolar disorder and psychotic depression), as well as for the control subjects.

Secondly, in order to statistically attest the uniqueness of the identified brain volume–cognition relationships for FEP subjects, we included “group status” as a further predictor variable in an analysis investigating the presence of significant correlations between GM volumes and cognitive performance across the FEP and control groups, searching for voxels where there were significant differences in the pattern of brain volume–cognition correlations between the two groups (with significant *F* values set at the *p* < 0.001 level, uncorrected for multiple comparisons). This analysis was restricted to the brain regions where significant findings had emerged in the single-group correlation analyses described above.

In all analyses, voxels of maximal statistical significance were converted from Montreal Neurological Institute (MNI) coordinates to the Talairach and Tournoux system using a simple MATLAB script, called mni2tal, written by Matthew Brett (MRC Cognition and Brain Sciences Unit, Cambridge, England).

## 3 Results

### 3.1 Separate brain volume–cognition relationships in the FEP group, diagnostic subgroups and control group

**Fig. 1:**
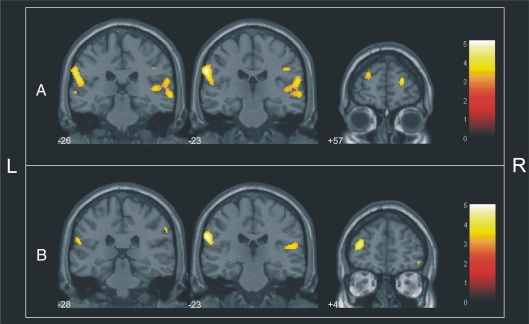
(A) Direct relationship between GM volume and cognitive performance in patients (first-episode psychosis group, *n* = 88) superimposed on coronal brain slices spatially normalized into an approximation to the Talairach and Tournoux stereotactic atlas. The numbers associated with each frame represent standard coordinates in the *y* axis. (B) Direct relationship between GM volume and cognitive performance in patients (schizophrenia subgroup, *n* = 48) superimposed on coronal brain slices spatially normalized into an approximation to the Talairach and Tournoux stereotactic atlas. The numbers associated with each frame represent standard coordinates in the *y* axis.

GM volumes were directly correlated (*p* < 0.05, FWE corrected for multiple comparisons) with cognitive performance in FEP patients (*n* = 88) in: the anterior portion of the superior dorsolateral prefrontal cortex on the right hemisphere (Brodmann's area — BA 10); the right inferior dorsolateral prefrontal cortex (inferior frontal gyrus; BA10/46); the lateral parietal cortex bilaterally (BA40); and the right superior temporal cortex (BA21/22/41/42) (Table 2, Fig. 1A). There were also trends towards direct correlations in: the anterior portion of the left superior dorsolateral prefrontal cortex (BA10, maximal *Z*-score = 3.64, coordinates_*x*__,*y*,*z*_ = − 24, 55, 21, *p* = 0.068, FWE corrected); and the left inferior dorsolateral prefrontal cortex, corresponding to the left middle frontal gyrus (BA46, maximal *Z*-score = 3.29, coordinates_*x*__,*y*,*z*_ = − 44, 36, 20, *p* = 0.059, FWE corrected). No significant direct correlations were detected in the exploratory investigation of other brain regions. There were no significant inverse correlations in this group.

In the schizophrenia subgroup (*n* = 48), the above trend towards a direct correlation within the left anterior dorsolateral prefrontal cortex (BA10) achieved statistical significance (Table 2, Fig. 1B). Significant direct correlations were also evidenced within the right inferior dorsolateral prefrontal cortex, corresponding to the right inferior frontal gyrus (BA47), the lateral parietal cortex bilaterally (BA40), and the left superior temporal cortex (BA41/42). There was also a trend towards a direct correlation in the right inferior dorsolateral prefrontal cortex (inferior frontal gyrus, BA46, maximal *Z*-score = 3.32, coordinates_x,y,z_ = 48, 39, 2, *p* = 0.056, FWE corrected). There was an unexpected inverse correlation in the posterior portion of the right lateral prefrontal cortex, encompassing the premotor portion of the middle frontal gyrus (BA6) (Table 2).

There were no significant direct or inverse correlations in the overall affective psychosis subgroup, in the bipolar disorder subjects or the psychotic major depression patients.

Control subjects (*n* = 86) showed unexpected foci of significant direct correlation within the right anterior dorsal cingulate gyrus (BA32), and clusters of inverse correlation in the parahippocampal gyrus bilaterally (BA30) (Table 2).

### 3.2 Interactions between brain volume–cognition relationships and group status

The inclusion of “group status” as a predictor variable in the analysis investigating correlations between GM volumes and cognitive performance across FEP patients and controls revealed one focus of significant interaction located in the left inferior dorsolateral prefrontal cortex corresponding to the middle frontal gyrus (BA46) (Table 3). There was an additional focus of significant interaction within the left parahippocampal gyrus (BA30) (Table 3).

These results indicated the uniqueness of the direct brain volume–cognition relationship within the left inferior dorsolateral prefrontal cortex for the FEP group, and the inverse brain volume–cognition relationship within the left parahippocampal gyrus for the control group.

The inclusion of “group status” as a predictor variable in the analysis investigating correlations between GM volumes and cognitive performance across the schizophrenia subgroup and controls revealed no foci of significant interactions.

### 3.3 Investigation of the influence of antipsychotic medication on GM volumes

As a large proportion of FEP patients were treated with antipsychotic agents at the time of testing, we conducted exploratory analyses comparing GM volumes between the two subgroups of medicated (*n* = 55) and unmedicated (*n* = 33) FEP patients. This analysis revealed no areas of regional GM volume differences between those subgroups, thus suggesting no influence of medication status on the patterns of significant cognition–brain volume described above.

## 4 Discussion

To the best of our knowledge, this is the first study that investigated the presence of GM volume–cognition relationships as assessed with VBM methods in a large, population-based sample of FEP subjects. Although the VBM approach showed volumetric abnormalities in several brain regions in this sample (Schaufelberger et al., 2007), direct GM volume–cognition correlations in FEP subjects were restricted to circumscribed dorsolateral prefrontal, lateral parietal and superior temporal cortical regions.

The FEP group displayed significant positive correlations with cognitive task performance in the dorsolateral prefrontal cortex, involving lateral BA10 and BA46; the interaction analysis including both FEP patients and controls (using “group status” as a predictor variable) showed that the brain volume–cognition relationship in left BA46 (middle frontal gyrus) was unique to the FEP group. These results reinforce the notion that the severity of cognitive deficits in psychosis may be greater in proportion to the degree of structural abnormalities in prefrontal regions (Bonilha et al., 2008; Gur et al., 1998; Salgado-Pineda et al., 2003). Our previously reported GM comparisons between FEP patients and controls (Schaufelberger et al., 2007) showed differences located in the same left inferior dorsolateral prefrontal region (BA 46) where we found direct GM volume–cognition correlations unique to the FEP group in the present study. The inferior dorsolateral prefrontal cortex, encompassing BA46, has been highlighted as a key prefrontal portion implicated in the pathophysiology of psychosis, from early stages of disease (Harrison et al., 2006; Molina et al., 2006). Also, BA46 is thought to play a major role in cognitive operations relevant to the neuropsychological tasks applied in the present study, involving executive functioning (Luna et al., 2002), attention (Johannsen et al., 1997) and verbal fluency (Abrahams et al., 2003) functions.

The fact that we found direct GM volume–cognition correlations in the FEP group also in a more anterior, right-sided dorsolateral prefrontal area (including lateral BA10), is consistent with the known heterogeneity of the prefrontal cortex in regard to cytoarchitecture, connectivity and function (Gilbert et al., 2006). The finding pertaining to BA10 could be related to our choice to investigate correlations between brain volume and neuropsychological performance using a summary index combining scores of separate cognitive tasks. A direct relationship with such a ‘synthetic’ aspect of cognitive function would be in line with the overall role of the anterior lateral prefrontal cortex in cognitive control (Koechlin et al., 2003). Lateral BA10 in particular, implicated in the current study, is thought to play a key role in executive functioning operations thought to be critical to the neuropsychology of psychosis (Gilbert et al., 2006).

We also found direct correlations between GM volumes in the lateral temporo-parietal neocortex bilaterally and cognitive performance in the FEP group. The parietal and temporal neocortices have been implicated in cognitive processes, such as language (Thomas et al., 1996), spatial working memory (Tek et al., 2002), and attention (Danckert et al., 2004). The lateral temporo-parietal sites are part of a brain network subserving semantic-lexical operations known to be impaired in psychosis (Thomas et al., 1996). Our results implicating the lateral temporo-parietal neocortex are consistent with previous findings of morphologic abnormalities of this region in ROI-based studies on chronic schizophrenia (Niznikiewicz et al., 2000), as well as with evidence of reduced activation of the left fronto-temporo-parietal cortex in association with language processing deficits in schizophrenia (Koeda et al., 2006).

When the correlation analyses were conducted separately for the schizophrenia subgroup, similar direct brain volume–cognition relationships as seen in the overall FEP sample emerged, although with a lesser degree of statistical significance (possibly due to the smaller sample size). The detection of significant direct correlations in the schizophrenia subgroup is consistent with neurodevelopmental models for the disorder (Murray and Lewis, 1987). According to such theories, early pathological processes in schizophrenia affecting the structural development of key brain regions are associated with cognitive deficits, which may precede the onset of full-blown psychotic symptoms (Stanfield et al., 2008).

Conversely, there were no significant correlations between GM volumes and cognitive indices in the affective psychosis subgroup. This difference is in contrast with our previous report of similar cognitive deficits in subjects with schizophrenia and affective psychoses in the population-based psychosis sample from which the groups described herein were drawn (Ayres et al., 2007). Thus although the neuropsychological profile of deficits may be similar across affective and schizophreniform psychoses (Albus et al., 1996; Hill et al., 2004; Zihl et al., 1998), the distinct neural substrate underlying cognitive deficits in each of these categories remains to be elucidated. However, it should be noted that our MRI study did not investigate structural brain abnormalities that might be more specifically related to the cognitive deficits associated with affective disorders, such as the white matter fasciculi that interconnect regions involved in the regulation of mood (Yurgelun-Todd et al., 2007). Also, the finding of significant correlations between brain volumes and cognitive measures only in the schizophrenia group may be due to the fact that there were substantially more patients with schizophrenia than with bipolar disorder or psychotic major depression in our overall psychosis sample. Therefore, our study may have lacked sufficient power to examine brain volume–cognition relationships in the subgroups of affective psychoses. Finally, differences in gender distribution (with a predominance of males in the schizophrenia subgroup, and females in the affective psychosis subgroup) may have also influenced on the emergence of cognition–brain volume relationships exclusively in schizophrenia subjects, as psychosis-related cognitive impairment is known to be more prominent in male individuals (Leung and Chue, 2000).

Within the prefrontal cortex, there was one unexpected site of significant inverse (rather than direct) correlation between GM volume and cognitive performance located in the posterior portion of BA6, in the schizophrenia subgroup. The diversity of findings involving the prefrontal region in our study is consistent with the notion that different prefrontal portions may exert distinct influences on cognitive functioning in psychosis. Also, it is possible that the correlation involving the premotor cortex might have been influenced by antipsychotic treatment, as we could not avoid the use of antipsychotic medication in a large proportion of our FEP sample. There are previous MRI findings suggesting that antipsychotic treatment may be associated with increased volumes of the premotor cortex in schizophrenia (Premkumar et al., 2008). However, we found no differences in GM volumes between our subgroups of medicated and unmedicated psychosis patients, thus arguing against any influences of medication status on the patterns of significant cognition–brain volume detected in this study.

Albeit scarce, the unexpected significant correlations obtained in the asymptomatic control group are potentially relevant, given that very few studies to date evaluated the relationship between grey matter volumes as assessed with VBM and cognitive indices in large groups of healthy volunteers (Tisserand et al., 2004). The finding of a direct correlation between cognitive performance and GM volume in the dorsal anterior cingulate cortex is consistent with the critical role attributed to this region in cognitive processing. The dorsal anterior cingulate cortex has been repeatedly implicated in cognitive control, including the allocation of attentional resources to support performance (Luks et al., 2002; Kerns et al., 2004), more specifically in response selection (Mesulam et al., 2001) with suggestions for monitoring the production of multiple responses (Carter et al., 1998; Fu et al., 2005). The fact that such correlation involving the anterior cingulate gyrus was not significant in the FEP group might relate to a greater degree of variability of attentional engagement in the FEP group relative to controls, possibly due to deficits in the functioning of the anterior cingulate region during task performance. Such possibility is supported by recent functional MRI findings of abnormal anterior cingulate functioning in schizophrenia subjects during the performance of cognitive tasks similar to those employed herein (Fu et al., 2005). It is relevant to note that any anterior cingulate aberrations in our FEP sample would have been present only at the functional level, as our previous comparison of grey matter differences in the psychosis group relative to controls revealed no macroscopic volumetric abnormalities in the former (Schaufelberger et al., 2007).

Also, there was an unexpected inverse correlation in the control group between cognitive performance and GM volumes in the parahippocampal gyrus, a brain structure also known to be relevant to the cognitive operations engaged by the tasks employed herein (Burgess et al., 2002; Seidman et al., 2003). Although this finding warrants replication in future studies, it is in accordance with the notion that in certain brain regions, there may be a relationship between better cognitive efficiency and lesser GM volume (possibly in association with lower synaptic density) (Frangou et al., 2004; Van Petten, 2004). In our study, there was statistical confirmation of the uniqueness of this correlation to the healthy control group and not FEP patients; this could be due to psychosis-related neuropathological processes in the hippocampal region affecting the relationship between cognitive performance and GM volumes in this region.

There are methodological aspects of our study that are distinctive from previous investigations. We recruited a sizeable, epidemiologically-based FEP sample, and with reduced risk of selection bias. Moreover, a large control group with no similar correlations to those present in the FEP group consolidates the findings detected in patients with schizophrenia. Finally, it has been well established that studies on the early course of psychosis favor the detection of biological features associated with the symptoms of the disorder itself and its associated cognitive deficits, with reduced confounding effects of long disease duration and chronic treatment (Flashman and Green, 2004; Saykin et al., 1994).

However, the results of cross-sectional studies such as ours must be interpreted with caution, as they do not necessarily imply a causal connection between anatomical deficits and cognitive impairment. Although the significant correlations most probably reflect localized GM deficits underlying cognitive impairment, one cannot discard the possibility that some of the foci of direct correlations might actually reflect better cognitive performance in association with larger GM volumes in regions not affected by the disorder. Prospective longitudinal studies combining MRI scanning and cognitive measures in FEP samples may further clarify such issues. In a recent longitudinal MRI study of first-episode schizophrenia (*n* = 20) studied twice over a 2–3 year period, patients exhibited progressive brain volume deficits despite improvements in neuropsychological performance, highlighting the probably complex relationship between brain structure and cognition in FEP (Zipparo et al., 2008).

The restriction of the cognitive battery to three readily applicable tests was necessary to allow the investigation of a large number of subjects in the parent, population-based from which the FEP and control samples described herein were drawn from (Ayres et al., 2007). The strategy of deriving one single summary measure of cognitive performance to be used for correlations with regional brain volumes was aimed to reduce the number of statistical tests performed, thus minimizing the risk of Type I errors. The use of a single, composite measure of psychosis-related cognitive dysfunction is also consistent with the view that psychotic disorders are characterized by a pattern of generalized cognitive impairment (Keefe et al., 2006; Dickinson et al., 2004). However, we acknowledge that our battery did not allow assessment of the broad range of cognitive functions that may be affected in FEP, possibly being insufficient to represent general cognitive ability. Nevertheless, there is extensive evidence that the three specific tasks employed herein validly estimate dysfunction on key cognitive domains in schizophrenia (Hutton et al., 1998; Riley et al., 2000; Silver et al., 2003).

In conclusion, our findings suggest that, as early as in the first episode of psychosis, cognitive performance is directly related to regional GM volumes, specifically in the dorsolateral prefrontal, superior temporal and lateral parietal cortices. Further imaging studies on this subject are warranted, including the assessment of subjects at risk for psychosis, as well as additional longitudinal MRI studies of FEP investigating the strength of GM volume–cognition correlations over the course of psychotic disorders.

## Role of funding source

Funding for this study was provided by the Wellcome Trust, UK. The Wellcome Trust had no further role in study design; in the collection, analysis and interpretation of data; in the writing of the report; and in the decision to submit the paper for publication.

## Contributors

All authors listed have contributed to all subsequent drafts, and have approved the final manuscript.

## Conflict of interest

None of the authors has conflicts of interest to report.

## Figures and Tables

**Table 1:** Sociodemographic data of first-episode psychosis group, first-episode schizophrenia subgroup, affective psychosis subgroup and control group.

Variables	First-episode psychosis group (*n* = 88)	Schizophrenia subgroup (*n* = 48)	Affective psychosis subgroup (*n* = 34)	Bipolar psychosis (*n* = 17)	Psychotic depression (*n* = 17)	Control group (*n* = 86)	Statistical significance (first-episode psychosis versus controls)	Statistical significance (schizophrenia subgroup versus controls)	Statistical significance (affective psychosis subgroup versus controls)
Gender (male)	52 (59%)	35 (73%)	14 (41%)	9 (52.9%)	5 (29.4%)	46 (53.5%)	0.46	0.027	0.23
Mean age (SD)	28.9 (8.6)	28 (8.4)	29.6 ± 8.6	28.2 (8.6)	31 (8.7)	30.6 (8.5)	0.13	0.06	0.52
Years of education^a^ (SD)	8.92 (3.6)	9.04 (3.3)	8.94 ± 3.96	8.3 (3.7)	9.5 (4.2)	10.15 (4.22)	0.045	0.09	0.21
Handedness (right/left/mixed)	79/7/2	43/4/1	30/3/1	17/0/0	13/3/1	83/2/1	0.082	0.108	0.084
Substance abuse or dependence	27 (30.7%)	17 (63%)	10 (29.4%)	7 (41.2%)	3 (17.6%)	5 (5.8%)	< 0.0001	< 0.0001	< 0.0001

Comparison of the three groups (first-episode psychosis group versus control subjects; schizophrenia subgroup versus control subjects; affective psychosis subgroup versus control subjects) on sociodemographic variables was performed with t tests, chi-square analyses. Group differences significant at p < 0.05. SD = standard deviation.

a Number of years of formal education.

**Table 2:** Brain regions showing significant correlations between GM volume and cognitive performance in separate analyses of first-episode psychosis subjects (*n* = 88), the schizophrenia subgroup (*n* = 48) and asymptomatic controls from the same geographical area (*n* = 86).

Anatomical location^a^	Search volume (voxels)	Cluster size^b^	Peak *Z*-score^c^	*p* value^d^	Talairach coordinates^e^		
*x*	*Y*	*z*
*Direct correlations*							
First-episode psychosis group (*n* = 88)							
Right superior dorsolateral prefrontal cortex (anterior portion; BA10)	9156	48	3.84	0.038	24	57	10
Right inferior dorsolateral prefrontal (BA10/46)	2072	19	3.46	0.034	48	39	4
Left lateral parietal cortex (BA40)	2296	72	4.09	0.005	− 57	− 26	18
Right lateral parietal cortex (BA40)	3137	278	3.95	0.010	61	− 23	16
Right superior temporal cortex (BA21/22/41/42)	3137	278	3.75	0.019	65	− 27	1
Schizophrenia subgroup (*n* = 48)							
Left superior dorsolateral prefrontal cortex (BA10)	8450	144	4.07	0.019	− 36	49	10
Right inferior dorsolateral prefrontal cortex (BA47)	2072	23	3.38	0.046	55	31	− 2
Left lateral parietal cortex (BA40)	2296	11	3.41	0.048	− 59	− 28	22
Right lateral parietal cortex (BA40)	3137	54	3.55	0.041	51	− 23	14
Left superior temporal cortex (BA41/42)	2296	11	3.41	0.048	− 59	− 28	22
Control group (*n* = 86)							
Right anterior cingulate gyrus (BA32)	1313	9	3.39	0.028	4	38	24

*Inverse correlations*							
Schizophrenia subgroup (*n* = 48)							
Right superior dorsolateral prefrontal cortex (posterior portion; BA6)	9156	130	3.93	0.033	24	− 5	57
Control group (*n* = 86)							
Left parahippocampal gyrus (BA30)	932	56	3.87	0.005	− 20	− 35	7
Right parahippocampal gyrus (BA30)	946	49	3.63	0.011	22	− 33	5

a Numbers refer to approximate Brodmann Areas (BA).

b Total number of contiguous voxels in each region above initial cutoff of *Z* > 3.09.

c *Z*-scores for the voxel of maximal statistical significance.

d Statistical significance after correction for multiple comparisons (voxel level).

e Talairach & Tournoux coordinates of the voxel of maximal statistical significance within each region.

**Table 3:** Differences between first-episode psychosis patients (*n* = 88) and healthy controls (*n* = 86) in the significance of correlations between GM volume and cognitive performance.

Anatomical location^a^	Cluster size^b^	Peak *F*-value^c^	Peak *Z*-score^d^	*p* value^e^	Talairach coordinates^f^			Direction of difference
*x*	*y*	*Z*
Left dorsolateral prefrontal cortex (middle frontal gyrus; BA46)	11	12.45	3.27	0.001	− 42	34	17	Greater positive correlation in FEP subjects relative to healthy controls
Left parahippocampal gyrus (BA30)	10	12.51	3.28	0.001	− 18	− 37	7	Greater negative correlation in healthy controls relative to FEP subjects

a Numbers refer to approximate Brodmann Areas (BA).

b Total number of contiguous voxels in each region above initial cutoff of *Z* > 3.09.

c *F*-values for the voxel of maximal statistical significance.

d *Z*-scores for the voxel of maximal statistical significance.

e Statistical significance uncorrected for multiple comparisons.

f Talairach & Tournoux coordinates of the voxel of maximal statistical significance within each region.
